# Lignin Nanoparticles for Enhancing Physicochemical and Antimicrobial Properties of Polybutylene Succinate/Thymol Composite Film for Active Packaging

**DOI:** 10.3390/polym15040989

**Published:** 2023-02-16

**Authors:** Angel Jr Basbasan, Bongkot Hararak, Charinee Winotapun, Wanwitoo Wanmolee, Wannee Chinsirikul, Pattarin Leelaphiwat, Vanee Chonhenchob, Kanchana Boonruang

**Affiliations:** 1 Department of Packaging and Materials Technology, Faculty of Agro-Industry, Kasetsart University, Bangkok 10900, Thailand; 2 National Metal and Materials Technology Center, National Science and Technology Development Agency, Pathum Thani 12120, Thailand; 3 National Nanotechnology Center, National Science and Technology Development Agency, Pathum Thani 12120, Thailand; 4 Center for Advanced Studies for Agriculture and Food, Kasetsart University, Bangkok 10900, Thailand; 5 Department of Horticulture, Faculty of Agriculture, Kasetsart University, Bangkok 10900, Thailand

**Keywords:** lignin nanoparticles, thymol, polybutylene succinate, antimicrobial packaging, biodegradable materials

## Abstract

The natural abundance, polymer stability, biodegradability, and natural antimicrobial properties of lignin open a wide range of potential applications aiming for sustainability. In this work, the effects of 1% (*w*/*w*) softwood kraft lignin nanoparticles (SLNPs) on the physicochemical properties of polybutylene succinate (PBS) composite films were investigated. Incorporation of SLNPs into neat PBS enhanced *T_d_* from 354.1 °C to 364.7 °C, determined through TGA, whereas *T_g_* increased from −39.1 °C to −35.7 °C while no significant change was observed in *T_m_* and crystallinity, analyzed through DSC. The tensile strength of neat PBS increased, to 35.6 MPa, when SLNPs were added to it. Oxygen and water vapor permeabilities of PBS with SLNPs decreased equating to enhanced barrier properties. The good interactions among SLNPs, thymol, and PBS matrix, and the high homogeneity of the resultant PBS composite films, were determined through FTIR and FE-SEM analyses. This work revealed that, among the PBS composite films tested, PBS + 1% SLNPs + 10% thymol showed the strongest microbial growth inhibition against *Colletotrichum gloeosporioides* and *Lasiodiplodia theobromae*, both in vitro, through a diffusion method assay, and in actual testing on active packaging of mango fruit (cultivar “Nam Dok Mai Si Thong”). SLNPs could be an attractive replacement for synthetic substances for enhancing polymer properties without compromising the biodegradability of the resultant material, and for providing antimicrobial functions for active packaging applications.

## 1. Introduction

The research of natural and biodegradable materials is of great interest, especially now that many industries, such as the packaging industry, are aiming for sustainability. Currently, biopolymers are the most attractive alternative for nonbiodegradable packaging materials, such as conventional plastics. However, previous studies have shown that biopolymers are still not equal to conventional plastics in terms of packaging performance.

Compared with conventional plastics, biopolymers have disadvantages, such as sensitivity to heat, humidity, and shear stress, that could lead to early partial thermal and mechanical degradation during the processing stage, and possess inferior mechanical properties—e.g., brittleness, rigidity, and low tensile strength—and inappropriate barrier properties—e.g., high water and oxygen permeabilities, resulting in limited application possibilities [1,2].

Innovations have been produced and a great amount research is ongoing to improve the functional performance and widen the applications of biopolymers. The most common and technically feasible method is adding another compound into the biopolymeric matrix to enhance material properties, such as mechanical, barrier, and thermal stability, and to provide active functions, including antimicrobial and antioxidant.

Recently, lignin has drawn distinct attention as a natural additive to polymeric matrices because of its capability to modify material properties. Lignin is a natural aromatic polymer exhibiting a complex, high molecular weight and a highly random structure. It is found in the cell walls of plants and constitutes the most stable component of biomass [3,4]. Lignin is an abundant agro-industrial waste, mainly from the papermaking and biorefinery industries. Its natural abundance and low cost open diverse potential applications in various industries. Lignin can be used in the agriculture industry as an agrochemical, i.e., as fertilizer, pesticide, and plant growth regulator [5]. In the medical industry, promising findings were reported, which may improve human health, involving the potential applications of lignin [6].

Aside from being naturally biodegradable, lignin generally shows high strength and rigidity and satisfactory thermal resistance. As a result, thermoplastic polymers compounded with lignin, such as polyethylene (PE), polypropylene (PP), and polyvinyl chloride (PVC), have shown improved flowability and processing performance of the material, and have exhibited significant enhancement in material properties [7]. In this present work, the effects of incorporation of softwood kraft lignin nanoparticles (SLNPs) on the properties of the resultant material were investigated. To be consistent with the aimed biodegradability and for the biopolymer composite not to be compromised, polybutylene succinate (PBS) was used as the polymer matrix. PBS is among the most important emerging biodegradable polymers, with a wide range of potential applications due to its excellent processability and chemical resistance [8]. It is synthesized by polycondensation between succinic acid and butanediol in a two-step reaction process: esterification between the diacid and the diol, and then polycondensation under high temperature to form PBS with high molecular weight [9]. Interestingly, PBS can be produced both from monomers derived from fossil-based sources and by the fermentation route of bacteria [10]. Moreover, it was reported that full biomass-based PBS was chemically synthesized from furfural that was produced from inedible agricultural cellulosic waste [11]. Even though PBS has excellent processability, some of its important properties, such as brittleness and low thermal stability, restrict its wide commercial implementation and applications. Hence, various techniques, such as copolymerization using the extrusion method coupled with a line for film casting [12], blending with another biopolymer through extrusion compounding followed by injection molding [13], and incorporation of natural additives [14], such as bamboo fiber, through extrusion followed by injection molding [15], have been performed to achieve improved properties. The thermal stability of the PBS polymer composite was enhanced with the addition of low sulfonate kraft lignin, but utilizing chemically modified low sulfonate lignin further improved the thermal stability of the resultant PBS composite [16]. It was reported that PBS and softwood kraft lignin have shown noncovalent interactions [17] which could possibly help in the enhancement of the thermal stability of the PBS composite. Contrasting results were presented when lignosulphonate (a classification of lignin) was blended in 70/30 PBS/polybutylene adipate-co-terephthalate (PBAT) with zinc nanoparticles, wherein the thermal stability and tensile strength of the hybrid composite decreased with the incorporation of lignosulphonate [18]. However, it is interesting to note that the lignosulphonate used in their study was not modified. In this present work, softwood kraft lignin was processed into nanoparticles to achieve the advantages of nano-size particles in terms of reactivity. Moreover, different classifications of lignin will produce different results when blended with a polymer, because the reaction is affected by the complexity of the lignin structure, which is dependent on the source and isolation process [19,20].

Aside from the effects that modify the properties of materials, lignin is a natural antimicrobial agent because it contains numerous functional groups [21] that are responsible for antimicrobial activities [22], such as phenolic and aliphatic hydroxyls, methyl, carboxylic, and carbonyl groups. However, despite its great potential to replace synthetic chemicals, only 2–5% of lignin in its macromolecular form is commercially utilized [23]. Considering that the worldwide paper and pulp industry discards lignin from its production, generating waste amounting to 50–70 million tons annually, the commercial utilization of lignin is considered very low.

Recent studies have shown the viability of lignin as an antimicrobial agent. For example, fractionated kraft lignin from bamboo obtained by organosolv fractionation can destroy, in vitro, the cell wall of *Escherichia coli*, *Salmonella*, *Streptococcus*, and *Staphylococcus aureus*, thus inhibiting the growth of these bacteria [24]. Similarly, it was reported that lignin derived from beechwood flour is comparable with existing antibacterial agents, such as chlorhexidine, in inhibiting the growth of *E. coli* and 189 *S. aureus* [25]. Moreover, lignin residue of corn stover from ethanol production exhibited antimicrobial activities against *Listeria monocytogenes*, *S. aureus*, and *Candida lipolytica* [26]. The authors [27] demonstrated that lignin from different sources, such as eucalyptus, acacia, sugarcane bagasse, corn, and cotton stalks, conferred antimicrobial activity against microorganisms mentioned above and several other microorganisms, including *Proteus vulgaris*, *Pseudomonas aeruginosa*, *Aspergillus niger*, *Bacillus subtilis*, and *Klebsiella pneumoniae*, among others.

In this present work, an innovative and sustainable manner of utilizing abundant agro-industrial waste lignin was presented by converting it into a high-value product that can be beneficial to the industry. Raw softwood kraft lignin was processed into lignin nanoparticles and used as a natural additive to enhance the material properties of PBS composite films. Additionally, while most previous published works dealt with bacteria, a potential novel work was demonstrated in this study by using the SLNPs as a natural antimicrobial agent against fungal species. *Colletotrichum gloeosporioides* and *Lasiodiplodia theobromae* are two major fungal species that cause significant postharvest decay—anthracnose and stem-end rot, respectively—to many economically important fruit crops such as mango, papaya, and citrus. The use of a natural additive, such as SLNPs, as an alternative to synthetic fungicides for postharvest decay control would not only be economically beneficial but also safer to humans and the environment.

The incorporation of essential oils into polymers as antimicrobial agents has widely been studied as a competitive alternative to chemical preservatives. Aside from being generally recognized as safe (GRAS), essential oils, which are hydrophobic liquids that are naturally present in various plant parts, demonstrate a wide range of antimicrobial activities, such as antibacterial and antifungal activities, and function as natural preservatives [28]. Their volatility enables them to diffuse out from the polymer matrix and perform antimicrobial functions, whereas the antimicrobial activities of SLNPs were restricted to the polymer matrices. The addition of SLNPs into polymer could enhance the antimicrobial efficacy of polymeric film. As thymol was found to be the most effective phenol against tested fungal species in our previous studies [29], SLNPs were added to thymol in the PBS composite. In our previous study, a high level of thymol, i.e., 20% (*w*/*w*), was used, whereas this present study aimed to utilize a lower amount of thymol with the addition of SLNPs.

Due to the reactivity of lignin as a function of the numerous functional groups found in it, lignin can interact with many polymers and change their properties [30]. According to this premise, SLNPs would produce interactions with PBS and thymol that could potentially enhance both the physicochemical and antimicrobial properties of the resultant PBS composite film. Therefore, this present work aimed to investigate the effects of the incorporation of SLNPs into PBS film containing thymol through characterizing the physicochemical properties of the resultant composite films in terms of morphology, Fourier-transform infrared spectroscopy (FTIR) analysis, and thermal, mechanical, and barrier properties. Moreover, the antimicrobial activities of SLNPs in PBS composite film containing thymol were investigated, both in vitro and in vivo.

## 2. Materials and Methods

### 2.1. Materials

Raw softwood kraft lignin powder (BioPiva^TM^100, denoted as SR-lignin) was purchased from UPM Biochemicals, Helsinki, Finland. PBS pellets were obtained from PPT Global Chemical Public Company Limited, Bangkok, Thailand. Pure culture of *C. gloeosporioides* was obtained from the Plant Protection Research and Development Office of the Department of Agriculture, Bangkok, Thailand, while pure culture of *L. theobromae* was isolated from “Nam Dok Mai Si Thong” mango fruit.

### 2.2. Preparation of SLNPs

SR-lignin was processed into lignin nanoparticles (LNPs) by antisolvent precipitation assisted by ultrasonication, following the method of Hararak et al. [31]. It was purified first through fractionation method using acetone. In detail, 10 g of SR-lignin was pre-dried in a vacuum oven at 80 °C for 6 h and then placed in a 3-neck flask containing 100 mL acetone. A water condenser was attached to the top neck of the flask while a thermocouple and nitrogen bubbling at a flow rate of 10 mL min^−1^ were attached to the other two necks. The flask containing the acetone and dried raw lignin was heated up, maintained at 56 °C, and then stirred for 6 h using a heating mantle with a built-in stirrer. The acetone-soluble fraction was filtrated through a 1 µm glass filter and the obtained acetone-soluble lignin (denoted as S-lignin) was dried at 80 °C in a vacuum oven for 30 min and then ground into powder.

The obtained S-lignin was processed into LNPs (denoted as SLNPs) by ultrasonication. Briefly, 1 g of S-lignin was fully dissolved in 20 mL acetone at room temperature. Afterward, the 20 mL acetone containing dissolved S-lignin was poured into 200 mL deionized water at room temperature under ultrasonication (VCX 500 sonicator, 500 watts, 20 kHz, and 13 mm probe) for 5 min. The mixture was then centrifuged at a speed of 11,180 g-force for 20 min and the obtained SLNPs were dried at 80 °C in a vacuum oven for 6 h and then kept in a hermetically sealed container until use.

### 2.3. Preparation of PBS Composite Films

In total, 2 masterbatches of polymer blends, namely, PBS + 2% SLNPs and PBS + 20% thymol, were initially prepared. The first masterbatch (MB) was prepared by mixing 2 g SLNP powder and 98 g PBS pellets and then gradually loaded the mixture into a co-rotating twin screw extruder (LABTECH Twin-screw extruder; 20 mm screw diameter; length to diameter ratio of 32). The barrel temperature profile from feeding to the die zone was set at 110, 130, 140, 150, 154, 155, 155, and 160 °C with a screw speed of 0.112 g-force. The produced extrudate passed through a cooling water, was air-dried, and then palletized to around 2.5 mm long. The second MB was prepared using an internal mixer (HAAKE™, Rheomex OS, Bremen, Germany; 310 cm^2^ capacity; 0.8 fill factor; roller rotor) to minimize the vaporization of the volatile thymol (vapor pressure of 53.33 Pa at 25 °C) and to produce good dispersion and homogeneity of the thymol in the PBS matrix [32,33]. The rotor speed was set at 0.038 g-force and the temperature of the mixing chamber was set at 120 °C. An amount of 250 g PBS pellets were gradually fed into the internal mixer until melt phase then a 62 g thymol powder was introduced into the mixer and allowed to mix for 12 min. The produced bulk polymer mixture was crushed into pellets. Proportions from the two masterbatches were pre-mixed to produce the final formulations, as reported in Table 1.

The mixtures were formed into composite films by using a blown film, single-screw extruder (Thermo Scientific HAAKE™ Rheomex OS, Bremen, Germany; 19 mm screw diameter; length to diameter ratio of 25) with a screw speed set at 0.038 g-force and temperature profile of 150, 155, 160, and 160 °C for the feeding, compression, metering, and die zone, respectively. Neat PBS film which served as the control for analyses was also formed using the same blown film, single-screw extruder with the same setting.

### 2.4. Physicochemical Characterization of PBS Composite Films

#### 2.4.1. Morphology

The morphology image of the cryofractured neat PBS and (selected) PBS composite films was carried out through a field-emission scanning electron microscope (FE-SEM, model SU5000, Hitachi, Tokyo, Japan). All samples were coated with platinum by Quorum-Q150RS (UK) before imaging.

#### 2.4.2. FTIR Analysis

The PBS composite films were determined for infrared absorption spectra using Fourier-transform infrared spectroscopy (FTIR, Tensor 27, Bruker Corporation, Bremen, Germany) with an attenuated total reflection (ATR) mode in the range of 500–4000 cm^−1^ with 64 scans.

#### 2.4.3. Thermal Properties

A differential scanning calorimetry (DSC, Mettler Toledo, Greifensee, Switzerland) was used to determine the glass transition temperature (*Ƭ_g_*) and the melting point temperatures (*T_m_*) of neat PBS and PBS composite films. The first heating scan was performed from −80 to 180 °C; the cooling scan was performed from 180 down to −80 °C; and the second heating scan was performed from −80 to 180 °C. The heating and cooling rates were set at 10 °C min^−1^, under a nitrogen atmosphere, with a flow rate of 50 mL min^−1^. From the data gathered in the DSC scan, the crystallinity of neat PBS and PBS composite films was calculated using Equation (1) [34]:

(1) $$\chi =\frac{\Delta H}{\Delta Ho(1-mf)}\;\times \;100$$

where *Δ*H is the heat of fusion of the sample obtained from the second heating scan, *Δ*H*_o_* is the heat of fusion of 100% crystalline PBS theoretically equal to 200 J/g [35], and (1 − *mf*) is the weight fraction of PBS in the composite films.

A thermogravimetric analysis (TGA, Mettler Toledo, TGA/SDTA 851e, Columbus, OH, USA) was used to determine the PBS composite films’ thermal decomposition (*Ƭ_d_*). Samples were heated from 30–600 °C at a heating rate of 10 °C min^−1^, under a nitrogen atmosphere, with a flow rate of 50 mL min^−1^. The remaining content of thymol in the composite films was also determined through the gathered data in TGA. It was calculated based on weight loss as a function of temperature.

#### 2.4.4. Mechanical Properties

The film samples were pre-conditioned inside a chamber set at 25 ± 2 °C and 50 ± 5% RH for 48 h. Young’s modulus (YM), tensile strength (TS), and elongation at break (EB) in machine direction were determined according to ASTM D882-09 using Instron universal testing machine (Model 5965, Instron, Norwood, MA, USA) equipped with 5 KN load cell and a cross-head speed of 10 mm min^−1^. At least 5 film samples (2.5 cm × 10 cm) were tested for each treatment.

#### 2.4.5. Barrier Properties

The oxygen transmission rate (OTR) was determined using an Oxygen Permeation Analyzer 8501 (Illinois Instruments, Inc., Johnsburg, IL, USA) in accordance with ASTM D3985-17. The oxygen permeability (OP), expressed in cm^3^ m h^−1^ m^−2^ atm^−1^, was calculated using the obtained OTR values (cm^3^ m^−2^ day^−1^), film thickness *l* (m), and difference in partial pressure between sides of the film *Δ*P (Pa), applied in Equation (2):

(2) $$\mathrm{OP}=\frac{\mathrm{OTR}\;\times \;l}{\Delta P}$$

The water vapor permeability (WVP) was determined using the desiccant method according to ASTM E96-95. Film samples were placed on metal cups filled with dehydrated silica gel. The water vapor transmission rate (WVTR, g m^−2^ day^−1^) was determined from the slope of the plot of the weight change of cup versus time using linear regression, divided by the area of diffusion (m^2^). The WVP (g m h^−1^ m^−2^ atm^−1^) was calculated using Equation (3) [32], using the determined WVTR, thickness *l* (m), and water vapor pressure difference *Δ*P (Pa) between sides of the film samples:

(3) $$\mathrm{WVP}=\frac{\mathrm{WVTR}\;\times \;l}{\Delta P}$$

### 2.5. Investigation of Antimicrobial Activities of SLNPs

#### 2.5.1. Isolation of Pure Culture of *L. theobromae*

The pure culture of *L. theobromae* was isolated from mango fruit cultivar “Nam Dok Mai Si Thong” following the method of Khan et al. [36]. Briefly, a small part of decaying mango fruit tissue near the stem end was cut using a sterile blade and placed on the potato dextrose agar (PDA) contained in Petri dishes. The Petri dishes were sealed with Parafilm and incubated in an ambient condition (30 ± 2 °C) until mycelial growth was observed. Subculturing was performed up to 3 times to isolate the pure culture of *L. theobromae*. Pure culture was verified using a light microscope and based on the colony and conidial characteristics.

#### 2.5.2. Antimicrobial Activities of SR-Lignin and SLNPs In Vitro

The poisoned food method, employed according to Balouiri et al. [37], was used to determine the antimicrobial activities using PDA. A mycelial plug of 8 mm diameter extracted from the edge of an actively growing, pure culture of *C. gloeosporioides* or *L. theobromae* was placed at the center of the newly prepared PDA containing SR-lignin or SLNPs. The final concentration of SR-lignin in PDA was 0.2% (*w*/*v*), while that of SLNPs varied from 0.2, 0.5, 2.0, to 5.0% (*w*/*v*). A pure PDA growth medium was used as the control. All prepared samples were sealed immediately with Parafilm and incubated at 30 ± 2 °C. Mycelial growth was measured each sampling day using a digital Vernier caliper (Mitutoyo 0.01 mm resolution) by measuring the average of the diameters in a perpendicular direction. The mycelial growth data were transformed into percentage inhibition using Equation (4) [38]. In total, 3 experiments with 3 replications per treatment were conducted for the determination of the antimicrobial activities of lignin in PDA.

(4) $$\mathrm{Inhibition}\;\left(\%\right)=100\;-\;\frac{Ø\;\mathrm{with}\;\mathrm{lignin}}{Ø\;\mathrm{control}}\;\times \;100$$

#### 2.5.3. Antimicrobial Activities of SLNPs Incorporated into PBS Composite Films In Vitro

The antimicrobial activities against *C. gloeosporioides* and *L. theobromae* of the SLNPs incorporated in PBS composite films containing thymol were determined using the vapor diffusion assay, according to Boonruang et al. [29], with slight modification. A film sample (7.5 cm diameter) was sterilized under UV light and then fixed inside the cover of a Petri dish containing solidified pure PDA inoculated with an 8 mm mycelial plug of pure culture of the tested fungal species. The antimicrobial activities were investigated at room temperature (30 ± 2 °C) and 12 ± 2 °C (optimum storage temperature for mango fruit). Neat PBS film served as the control. The incubation condition, measuring of mycelial growth, and calculation of percentage inhibition were the same as described in Section 2.5.2.

#### 2.5.4. Antimicrobial Activities of PBS Composite Films In Vivo

The antimicrobial activities of the developed PBS composite films were tested on mango fruit (*Mangifera indica* cultivar “Nam Dok Mai Si Thong”). Export quality mangoes (85% maturity, 100 days from full blossom) were purchased from Chiang Mai, Thailand, and carefully delivered to the laboratory. The mango fruit were sorted further based on similarity in shape, size, color peel, and absence of visual defects. The selected mango fruit were washed with tap water, sanitized with 200 ppm NaOCl for 2 minutes, and then air-dried for 4 h at ambient room temperature before being packed in neat PBS, PBS + 10T, and PBS + 1SLNPs + 10T composite films (20 cm × 15 cm), with 3 samples each. Mango fruit without packaging served as the control. The prepared samples were stored in a cold chamber set at 12 ± 2 °C and 90 ± 5% RH. The total area of decay caused by both *C. gloeosporioides* and *L. theobromae* was quantified by measuring the average perpendicular diameters of the decay at the end of the storage time, using a digital Vernier caliper (Mitutoyo 0.01 mm resolution).

### 2.6. Statistical Analysis

SPSS Statistics was used to perform an analysis of variance (ANOVA) test on samples. Duncan’s multiple range test (DMRT) was employed to determine differences among sample means at a significant level of *p* ≤ 0.05.

## 3. Results

### 3.1. Physicochemical Characterization of PBS Composite Films

#### 3.1.1. Morphology

**Figure 1:**
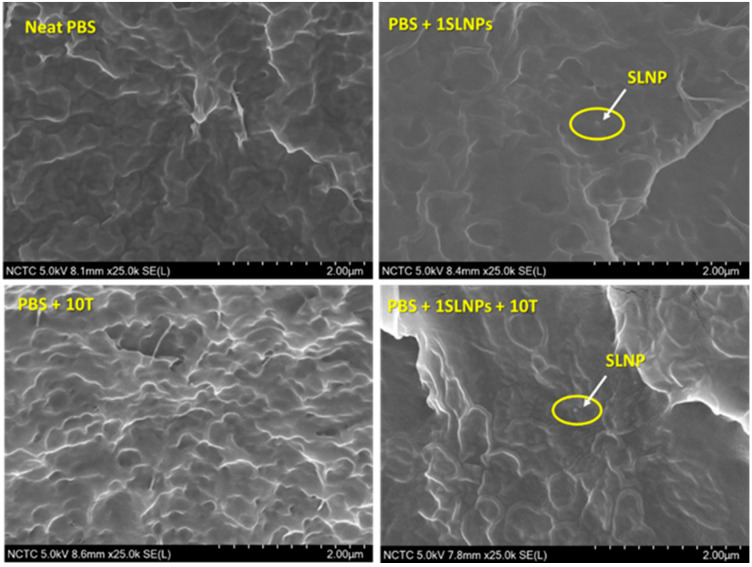
SEM images of cryo-fractured cross-section (side view) of neat polybutylene succinate (PBS) and PBS composite films with thymol (T) at a magnification of ×25 K. The size of softwood kraft lignin nanoparticles (SLNPs) ranged from 40–300 nm with an average particle size of 120 ± 18 nm and average polydispersity index (PDI) of 0.07 ± 0.01, determined by dynamic light scattering (DLS) technique at 25 °C (Malvern Zetasizer-4 Instrument, Malvern, UK).

Figure 1 shows the cryo-fractured cross-section image of neat PBS, PBS + 10T, PBS + 1SLNPs, and PBS + 1SLNPs + 10T films. The captured image of the neat PBS film showed a relatively smooth cross-section surface with fibrils forming web-like structures, indicating a semi-ductile characteristic of the PBS polymer [39]. The smoothness of the cross-section surface remains when 10% thymol was added into the PBS matrix. Furthermore, no visible phase separation was seen on the image with the presence of 10% thymol in the PBS + 10T composite film, an indication of the homogeneity of the PBS composite. The incorporated 1% SLNPs were very small relative to the PBS matrix; hence, only a single particle of the SLNPs was captured in the images of both PBS + 1SLNPs and PBS + 1 SLNPs + 10T. Moreover, no agglomeration of SLNPs was seen, an indication of good dispersion of the 1% SLNPs in the PBS matrix.

#### 3.1.2. FTIR Analysis

**Figure 2:**
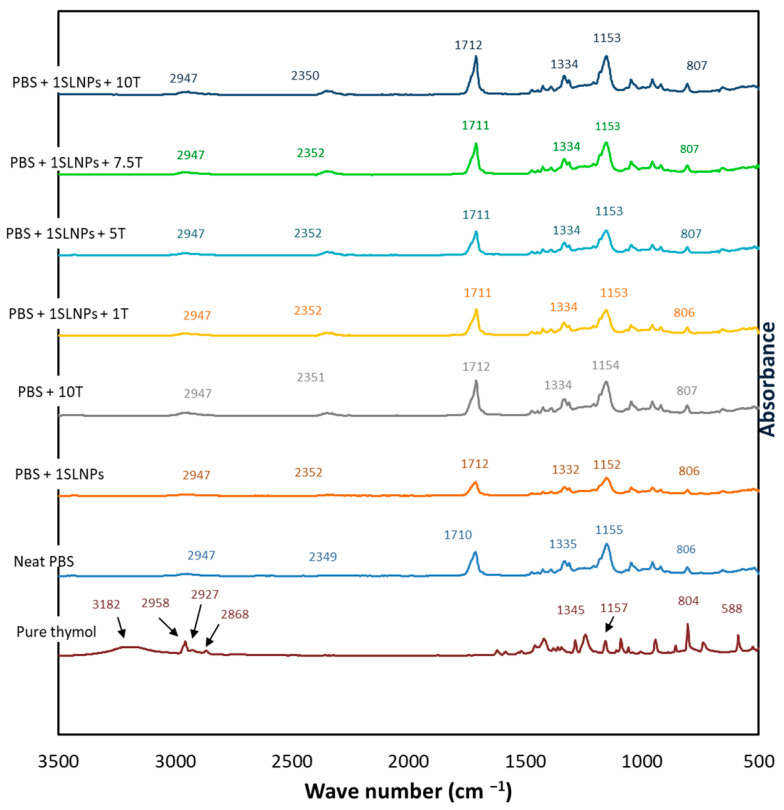
FTIR spectra of neat PBS, pure thymol, and PBS composites.

**Figure 3:**
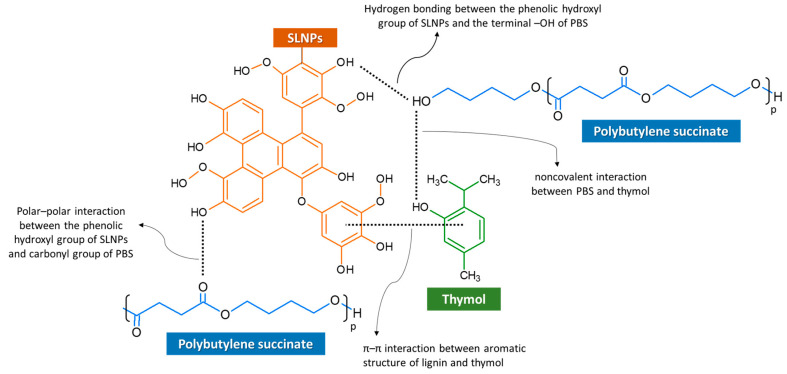
Representation of interactions among SLNPs, thymol, and PBS.

Interactions among 1% SLNPs, thymol, and PBS matrix were identified using ATR-FTIR analysis. Figure 2 shows the IR spectra of pure thymol, neat PBS film, and PBS composite films. The FTIR curves of the PBS composite films with 1% SLNPs + varied concentrations of thymol showed a similarity of patterns, indicating a high degree of homogeneity of the films. This could be attributed to the occurrence of intermolecular interactions, such as hydrogen bonding, between the phenolic hydroxyl groups in SLNPs and the terminal –OH or –COOH of PBS, π–π interactions between aromatic structures of lignin and thymol, and possible polar–polar interactions between the phenolic hydroxy group of SLNPs and carbonyl group of PBS [40] (Figure 3). The peaks at 2958–2868 cm^−1^ were ascribed to the symmetric and asymmetric stretching of C–H in thymol [41]. Meanwhile, the neat PBS film and all PBS composite films showed CH_2_ vibration of the methylene group at 2947 cm^−1^, which is typical for PBS [39]. The observed peak at 2349 cm^−1^ was assigned to asymmetric stretching of O=C=O, the peak at 1710 cm^−1^ was ascribed to C=O stretching of the carboxyl group, and the peak at 1335 cm^−1^ occurred due to C–O stretching of the ester group [42] in neat PBS. These peaks shifted slightly, to 2350–2352 cm^−1^, 1711–1712 cm^−1^, and 1332–1334 cm^−1^, respectively, signaling interactions among 1% SLNPs, thymol, and PBS matrix. The broadening of the peak at 1152 cm^−1^ for PBS containing 1% SLNPs and thymol lower than 10% concentrations was due to the generation of intermolecular hydrogen bonding and Van der Waals force among PBS, SLNPs, and thymol [42]. Moreover, the observed 1155 cm^−1^ peak in neat PBS film, which was attributed to the ester C–O–C bond stretching [43], showed a slight change of wavenumbers, to 1154 and 1153 cm^−1^, when 10% thymol (PBS + 10T) and 1% SLNPs (PBS + 1SLNPs + 10T) were added, respectively. The slight shift implied that there were some noncovalent interactions between PBS and thymol [17], and possibly among PBS, SLNPs, and thymol. The intense band spectra ring vibration of thymol seen at 804 cm^−1^ was attributed to out-of-plane C–H wagging vibrations, and the slight shifting of this band, to 806–807 cm^−1^, indicated the presence of thymol in the PBS composites [41]. Meanwhile, the presence of intense bands of thymol at 588 cm^−1^ was ascribed to O–H out-of-plane deformation vibration of the content phenols [44].

#### 3.1.3. Thermal Properties

The thermal properties of neat PBS film and PBS composite films were reported in terms of glass transition temperature (*Ƭ_g_*) and melting temperatures (*T_m1_* and *T_m2_*), determined through DSC analysis, and decomposition temperature (*Ƭ_d_*), determined with the use of TGA. The results of the DSC analysis were obtained from the second heating scan of film samples, because this provided data on the ideal behavior of the material, since residual solvents and the thermal history of the material were removed in the first heating scan. Thermal properties and crystallinity of the neat PBS film and PBS composite films are summarized in Table 2. The *Ƭ_g_* of the neat PBS film was determined to be −39.1 °C, and the addition of 1% SLNPs increased the *Ƭ_g_* to −35.7 °C. The increase in *Ƭ_g_* was an indicator of good interactions between 1% SLNPs and the PBS matrix as a result of strong intermolecular hydrogen bonds between polymers [45,46]. Meanwhile, the 10% (*w*/*w*) thymol functioned as a plasticizer to the PBS matrix, decreasing the *Ƭ_g_* of the PBS + 10T film to −43.7 °C. The plasticizing effects of essential oils, such as thymol, can reduce the intermolecular interactions of the polymer chains, increasing mobility and leading to improved flexibility and ductility of the polymer [33,47,48]. The lower amount of thymol, i.e., 1 and 5% (*w*/*w*), had insignificant plasticization effects in the PBS + 1SLNPs + 1T and PBS + 1SLNPs + 5T films, since the *Ƭ_g_* increased to −33.2 and −34.3 °C, respectively. However, it was observed that the *Ƭ_g_* in PBS + 1SLNPs + 10T films decreased to −42.1 °C, owing to the plasticization effects of the higher amount of thymol in the PBS matrix. It was previously believed that lower thymol concentrations, i.e., of 1 and 5%, had no effect on the intermolecular bond between 1% SLNPs and PBS, yet it was observed that these concentrations produced a non-plasticizing effect in the PBS composites.

**Figure 4:**
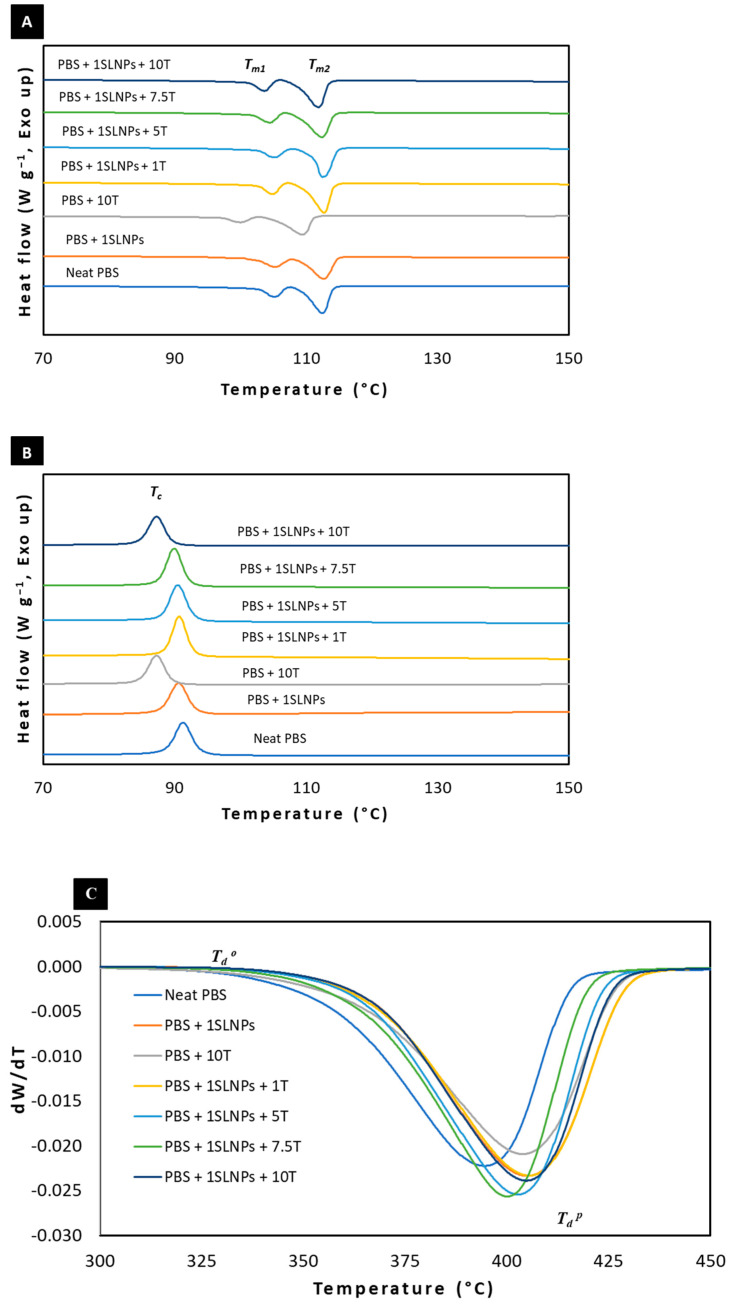
DSC thermograms of neat PBS and PBS composite films under nitrogen atmosphere—(**A**) second heating scan melting peaks and (**B**) crystallization peak; (**C**) derivative thermogravimetry of neat PBS and PBS composite films showing the onset and peak of thermal decomposition using TGA.

The melting peaks of neat PBS and PBS composite films are shown in Figure 4A. As illustrated, neat PBS and PBS composite films exhibited double melting peaks—*T_m1_* and *T_m2_*—due to the two different types of crystalline lamella present in PBS. The lower melting exotherm (*T_m1_*) corresponded to the melting of the original crystallites, while the higher melting exotherm (*T_m2_*) revealed the melting of the recrystallized crystals [39]. Generally, the *T_m_* increases with the increase in crystallinity [49], due to the higher energy required for melting. The presence of 1% SLNPs in the PBS matrix had no significant effects both on the *T_m1_* and *T_m2_* of the PBS composites, the same as thymol <10%. This was reflected in the % crystallinity, where the calculated values showed no significant change. However, PBS composite films with 10% thymol, either with or without 1% SLNPs, showed noticeably reduced melting temperatures. The present work was in accordance with our previous study [33] which revealed that incorporation of 10 to 20% (*w*/*w*) thymol into antifungal PLA films led to the depletion of the *T_m_* from 150 °C (neat PLA film) to 142 °C (PLA + 10% thymol film). Similar results were reported by Celebi and Gunes [50], where neat PLA and PLA containing 5% thymol had the same *T_m_* at 154 °C, but the *T_m_* decreased to 148 °C with 10% thymol in the polymer matrix. They explained that, due to the low molecular sizes of the plasticizers, thymol occupied the intermolecular spaces between the polymer chains, resulting in (1) reduced energy for molecular motion, (2) decreased formation of hydrogen bonding between polymer chains, and (3) increased free volume and molecular mobility. Nevertheless, comparing the 2 PBS composite films, PBS + 10T had lower *T_m1_* and *T_m2_* (100 and 109.5 °C, respectively) than PBS + 1 SLNPs + 10T (103.7 and 111.8 °C, respectively). It could be stated that the 1% SLNPs in the PBS composite helped to lessen the reduction of hydrogen bonding between PBS chains caused by the plasticization effects of thymol.

The *T_c_* is the temperature where the molecules have enough energy to form into ordered arrangements [51]; therefore, certain molecules crystallize at this temperature [52]. Figure 4B shows the *T_c_* observed in the cooling scan of the DSC thermogram, and the analyzed values are summarized in Table 2. Similar to the obtained results for the melting temperatures, the 1% SLNPs and thymol <10% had no significant effects on the *T_c_* of the PBS composites. It was noticeable, however, that the lowest *T_c_* was obtained in PBS + 10T at 87.5 °C; however, with the presence of 1% SLNPs, the obtained *T_c_* was increased to 89.7 °C.

Thermal stability of neat PBS and PBS composites films was analyzed through TGA by determining the onset and peak of the *Ƭ_d_* under a nitrogen atmosphere. The thermal stability of a polymer refers to the capability of the polymer to resist thermal action while maintaining its properties (strength, toughness, elasticity) at a given temperature [53]. Figure 4C shows the derivative thermogravimetry of neat PBS and PBS composite films; the onset and peak of *Ƭ_d_* is shown. The onset of *Ƭ_d_* was recorded at 354.1 °C for the neat PBS film. Incorporation of 1% SLNPs into PBS increased the onset *Ƭ_d_* to 364.7 °C, and the incorporation of 10% thymol increased the *Ƭ_d_* to 361.7 °C. The results showed that 1% SLNPs in the PBS enhanced the thermal stability of the PBS matrix more than the 10% thymol. Moreover, the combined effects of 1% SLNPs and 10% thymol in the PBS matrix enhanced further the thermal stability of the resultant PBS composite film, in which the recorded onset of *Ƭ_d_* of PBS + 1SLNPs + 10T was 365.2 °C. Furthermore, as an effect of the presence of 1% SLNPs in the PBS composites, the recorded onset of *Ƭ_d_* of PBS + 1SLNPs + T was 364.6, 362.7, and 362.9 °C for 1T, 5T, and 7.5T, respectively, which were generally higher than the onset of *Ƭ_d_* of PBS + 10T (without 1% SLNPs). The peak of *Ƭ_d_* revealed that 1% SLNPs could improve the *Ƭ_d_* of PBS composite more than 10% thymol. Moreover, the peak of *Ƭ_d_* revealed that thymol could help to improve the *Ƭ_d_* of PBS composites, but the incorporation of 1% SLNPs into PBS + 10T could enhance the *Ƭ_d_* to a greater degree. The obtained results showed that 1% SLNPs and thymol both enhanced the thermal stability of PBS, a finding that is in accordance with Mariën et al. [54] and Mousavioun et al. [55], who reported that lignin fragments strongly improved the thermal stability of modified silicone polymer and soda lignin improved the overall thermal stability of poly(hydroxybutyrate) or PHB, respectively. Meanwhile, Mohamad et al. [42] reported that the incorporation of thymol into PBS could help enhance the thermal stability of neat PBS.

Based on the TGA results, the remaining content of thymol in PBS + 1SLNPs + 1T, PBS + 1SLNPs + 5T, and PBS + 1SLNPs + 7.5T composite films was 0.99, 4.96, and 7.45%, respectively. Meanwhile, the remaining content of thymol in PBS + 10T was 9.58%; this was slightly increased to 9.72% in PBS + 1SLNPs + 10T, which could be an effect of the π–π interactions between aromatic structures of SLNPs and thymol in the PBS matrix (Figure 3).

The degree to which the polymer chains are aligned with one another is referred to as the crystallinity of a polymer [56]. The calculated % crystallinity of the neat PBS and PBS composites as affected by 1% SLNPs and thymol conformed to the obtained *Ƭ_g_*, *T_m_*, and *Ƭ_d_* of the composite materials. As shown in Table 2, the % crystallinity of neat PBS was 36.1%, and was slightly increased to 38.5% when 1% SLNP was incorporated into the PBS matrix, due to the nucleation effects of lignin. Lignin can act as a nucleating agent [57,58]; hence, it can increase the crystallinity of a polymer. A similar result was reported by Sahoo et al. [45], who concluded that the % crystallinity of PBS increased with the addition of lignin up to 65% (*w*/*w*)—a much higher amount of lignin compared with the present study. Moreover, 10% thymol caused a decrease in the % crystallinity of PBS + 1SLNPs + 10T film, which confirmed the plasticizing effects affecting *Ƭ_g_* and *T_m_* of the PBS composite. This is in accordance with our previous study which showed the reduction of the degree of crystallinity of PLA after the addition of 10–20% (*w*/*w*) thymol [33]. A similar result, that PBS blended with 2–10% (*w*/*w*) thymol exhibited a lower degree of crystallinity compared with neat PBS, was reported by [59].

#### 3.1.4. Mechanical Properties

The mechanical properties of a material describe its behavior upon the application of external force(s). Table 3 shows the effects of 1% SLNPs, thymol, and combined 1% SLNPs and thymol on the Young’s modulus (YM), tensile strength (TS), and elongation at break (EB) of the PBS composite films.

The YM conformed to the *T_m_* of the film samples, in general. PBS composites with higher *T_m_* showed higher YM. This relationship is associated with molecular mobility as a result of the strength of the atomic bonding of polymers in the composite [60]. Lignin is highly interactive, which can enhance the films’ mechanical properties, due to the strong hydrogen bonds between its functional group and the polymer matrix [61]. This could be the reason for the increase in YM from 593.4 to 601.9 MPa, and in TS from 34.3 to 35.6 MPa, when 1% SLNP was incorporated into the PBS matrix. Similarly, the YM and TS of wheat gluten bioplastic increased with the presence of kraft lignin in the polymer, as reported in [62]. Sakunkittiyut et al. [63] showed that up to 30% (*w*/*w*) of kraft lignin significantly increased the YM and TS of a fish protein-based polymer by 50 and 300%, respectively. Moreover, the YM and TS of starch/lignin bio-composites increased with the addition of a concentration of lignin up to 3% (*w*/*w*) [64]. Similarly, the YM and TS of agar/lignin bio-composites increased [65], and the YM and TS of whey protein isolate/lignin bio-composites increased with the addition of a 0.5% (*w*/*w*) concentration of lignin [66]. Meanwhile, it was expected that the YM and TS of the PBS composites containing thymol would generally decrease, as thymol lessens the strong intermolecular bonding of PBS chains, resulting in higher mobility of the chain [59,67]. These studies dealt with a low level of lignin, from 0.5 to 3.0%, to attain an improved YM and TS. Other previous studies revealed that a higher amount of lignin in the polymer matrix resulted in decreased YM and TS. Aadil et al. [68] reported that high lignin concentrations, of 10 and 20%, decreased the TS of a lignin–alginate composite down to 0.5 and 0.1 MPa, respectively, compared to the TS of a neat alginate, which was 0.6 MPa. A similar result was shown by Izaguirre et al. [69]; lignin concentrations higher than 0.25% (*w*/*w*) did not increase further the YM and TS of chitosan films. The decrease in YM and TS of the polymer matrix incorporated with a higher level of lignin could be the result of poor interfacial interaction between the lignin and the polymer matrix, due to agglomeration of lignin particles in higher concentrations caused by strong hydrogen bonding between the functional groups present in lignin [70].

The use of 1% SLNPs in the present work seemed to be the appropriate level, as it enhanced the mechanical properties, in particular, the YM and TS, of the PBS composite films. For the EB, the results showed that the change was not significant (*p* ≤ 0.05) as compared to the neat PBS.

#### 3.1.5. Barrier Properties

Barrier properties of the neat PBS and PBS composite films are reported in terms of OP and WVP (Table 4). The calculated OP of neat PBS was 2.28 × 10^−3^ cm^3^ m h^−1^ m^−2^ atm^−1^ and significantly reduced to 1.59 × 10^−3^ cm^3^ m h^−1^ m^−2^ atm^−1^ with the incorporation of 1% SLNPs. This effect was due to the creation of a winding path by the lignin in the PBS matrix, resulting in difficulty for the movement of the diffusing O_2_ molecules. The O_2_ molecules navigated around the lignin dispersed in the PBS, resulting in a longer time of diffusion [71]. The reduced OP of the PBS + 1SLNPs was related to the increased crystallinity of the PBS composite films. Since lignin is a nucleating agent [57], the presence of the SLNPs in the PBS matrix could result in the development of crystal phases that are assumed to be impermeable [72]; this could hinder the movement of the diffusing O_2_ molecules. Moreover, the reduced OP of PBS with the presence of 1% SLNPS could be due to the reinforcing effects of lignin on the biobased polymer matrix. The results were in accordance with Kovalcik et al. [73], who reported that the OP of poly(3-hydroxybutyrate-co-3-hydroxyvalerate) (PHBHV) bio-polyester decreased with the addition of 1% (*w*/*w*) kraft lignin, from 6.97 × 10^−4^ cm^3^ m h^−1^ m^−2^ atm^−1^ down to 1.69 × 10^−4^ cm^3^ m h^−1^ m^−2^ atm^−1^. The effectiveness of the 1% SLNPs in reducing the OP of the PBS composite films can also be associated with the rigid nature of lignin particles in conjunction with the good dispersion of the SLNPs in the PBS matrix.

Meanwhile, the general increase of the OP of the PBS matrix incorporated with thymol was ascribed to the destabilization of the PBS chain due to the plasticizing effects of thymol leading to a less dense and less cohesive PBS polymer network (in the case of PBS + 10T) and reduced intermolecular interaction between SLNPs and PBS chain (in case of PBS + 1SLNPs + varied concentrations of thymol). The increased OP values in PBS composites with thymol were probably due to the changes in the PBS matrix structure caused by thymol, as proven by the FTIR analysis. These changes increased the diffusion of O_2_ molecules through the PBS composite films with thymol. This was consistent with Othman et al. [74], who reported that the OP values of corn starch films increased as an effect of thymol present in the polymer matrix.

The WVP of the neat PBS reduced from 1.54 × 10^−3^ g m h^−1^ m^−2^ atm^−1^ to 1.41 × 10^−3^ g m h^−1^ m^−2^ atm^−1^ with the addition of 1% SLNPs because of the difficult path for the water vapor in the PBS matrix created by the lignin. This reduction could also be due to the good compatibility between 1% SLNPs and the PBS matrix leading to strong molecular interactions between polymers, as shown in Figure 2 and Figure 3. Previous studies have shown that lignin enhanced the water vapor barrier of thermoplastic starch [75], sago starch-based food packaging film [64], agar composite films [65], and soy protein isolate film [30]. Meanwhile, the addition of 10% thymol in the PBS caused a significant decrease in the WVP of the PBS composites. This reduction in WVP was due to the hydrophobic property of thymol resulting in decreased affinity of the PBS composite with water. Moreover, the hydrogen and covalent interactions between the PBS network and the phenolic compound of thymol may reduce the availability of hydrogen groups to produce hydrophilic bonds with water [74].

### 3.2. Investigation of Antimicrobial Activities of SLNPs

#### 3.2.1. Antimicrobial Activities of SR-Lignin and SLNPs In Vitro

**Figure 5:**
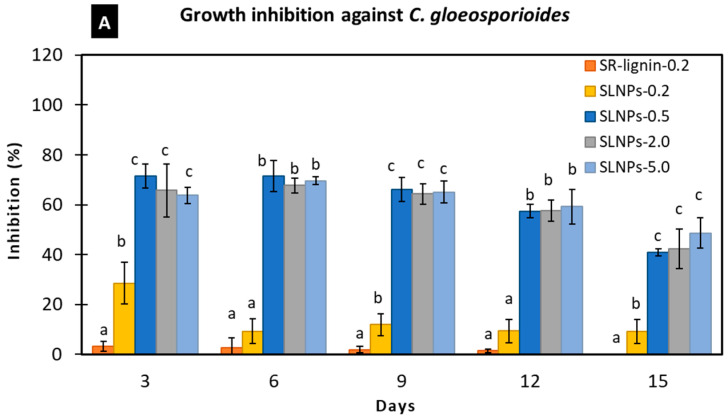

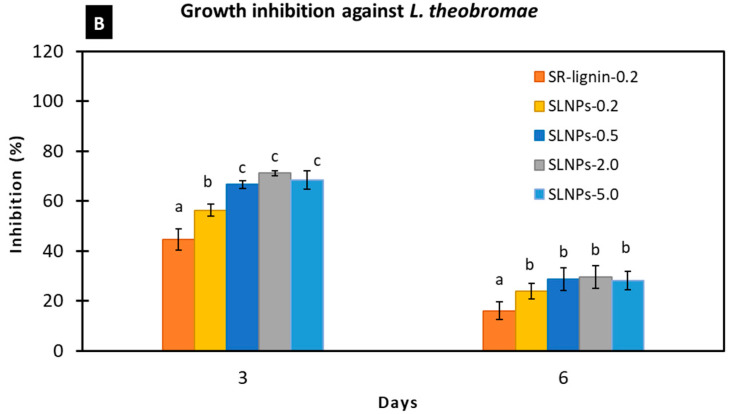
Antimicrobial effects of raw softwood kraft lignin (SR–lignin) and SLNPs in PDA against (**A**) *Colletotrichum gloeosporioides* and (**B**) *Lasiodiplodia theobromae*. Values are presented as mean (*n* = 3) with the standard deviation represented by vertical bars. Means followed by the same letter between treatments per day are not significantly different at *p* ≤ 0.05 using DMRT. SR–lignin: raw softwood kraft lignin.

The poisoned food method is a common technique used to evaluate the antimicrobial activities against fungi. In this method, the antimicrobial agent is mixed well into the growth medium at the desired final concentration [37]. The present study investigated the antimicrobial activities of SR-lignin and SLNPs against *C. gloeosporioides* and *L. theobromae* through the poisoned food method, at 30 ± 2 °C, using PDA as the growth medium. As shown in Figure 5A, the SR-lignin at 0.2% (*w*/*v*) concentration showed low growth inhibition against *C. gloeosporioides*, with 2.03, 4.03, 1.26, and 0.70% on days 3, 6, 9, and 12, respectively. There was no inhibition on day 15 because the mycelia on both SR-lignin samples and control reached the edge of the Petri dish. It was found that purifying the SR-lignin and processing it into LNPs significantly increased the antimicrobial activities, to 646.79%, at the same concentration of 0.2% (*w*/*v*). Moreover, the growth inhibition was significantly increased further when the concentration of SLNPs was increased from 0.2 to 0.5% (*w*/*v*). The growth inhibition of SLNPs at 0.5% (*w*/*v*) concentration was 352.06% higher than SLNPs at 0.2% (*w*/*v*) concentration. Therefore, it was expected that a concentration of SLNPs higher than 0.5% (*w*/*v*) would result in a stronger growth inhibition. However, the growth inhibition against *C. gloeosporioides* in 0.5, 2.0, and 5.0% (*w*/*v*) concentrations were not significantly (*p* ≤ 0.05) different throughout the days of observation. The results were comparable with what was reported by Dominguez-Robles et al. [17], regarding the degree of adherence of *S. aureus* on the PBS composites containing different concentrations of lignin. PBS composites containing 2.5, 5, 10, and 15% (*w*/*w*) lignin showed a similar degree of bacterial adherence at *p* ≤ 0.05, which suggests that the resistance to bacterial adherence was not directly dependent on lignin concentration.

**Figure 6:**
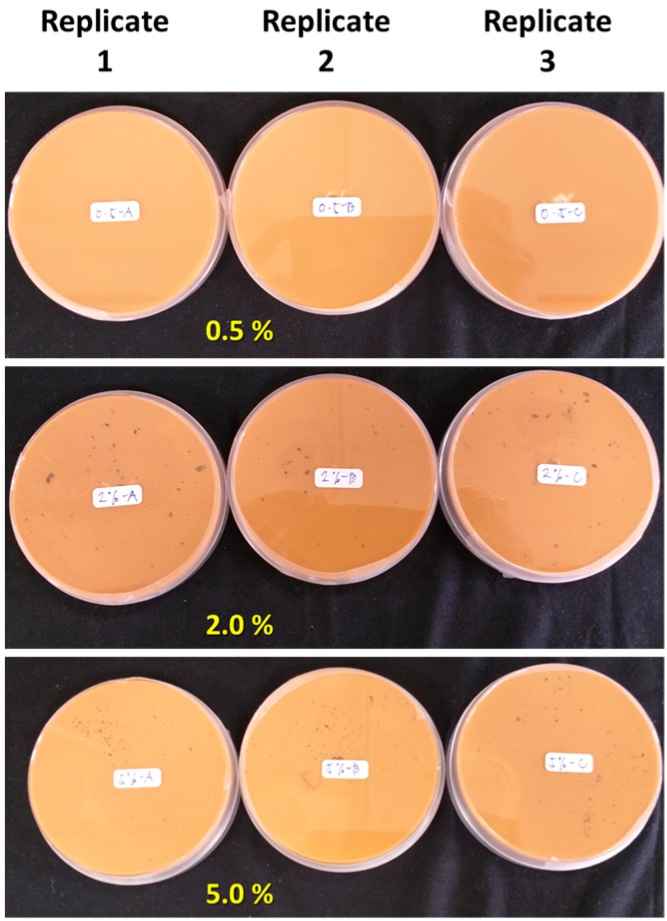
Reverse side of the Petri dishes containing PDA growth medium. The black spots visible at 2.0 and 5.0% were agglomerated SLNPs.

SR-lignin at 0.2% (*w*/*v*) concentration showed a stronger growth inhibition against *L. theobromae*, as shown in Figure 5B. The growth inhibitions were 44.61 and 16.07% on days 3 and 6, respectively. With the same concentration of 0.2% (*w*/*v*), SLNPs showed 32.06% significantly stronger antimicrobial effects compared with SR-lignin. With an increase in the SLNPs’ concentrations from 0.2 to 0.5% (*w*/*v*), the growth inhibition significantly increased, by 18.96%, and further increasing the concentrations, to 2.0 and 5.0% (*w*/*v*), produced no significant (*p* ≤ 0.05) increase in the antimicrobial effects. The same level of growth inhibition at 0.5, 2.0, and 5.0% (*w*/*v*) concentrations could be due to the agglomeration of SLNPs which occurred, visible on the reverse side of the Petri dishes containing the growth medium (Figure 6). The agglomeration of SLNPs at higher concentration can hide a portion of the surface area of the SLNPs, affecting the full interactions against the tested fungal species. The agglomeration could be the result of high surface energy, a large number of hydrogen bonds, and Van der Waals forces between SLNP particles [76]. In comparison, the growth inhibition of SR-lignin and SLNPs, both at 0.2% (*w*/*v*) concentration, was higher than that of *C. gloeosporioides* against *L. theobromae*. However, it could be observed that the antimicrobial effects against *L. theobromae* remained up to 6 days, compared with the effects against *C. gloeosporioides*, which persisted for 15 days. The difference in the antimicrobial activities was the outcome of the faster growth of *L. theobromae* at 30 °C [77].

The presence of SLNPs in the culturing media could inhibit growth and cause changes in the morphology of the microorganism. In particular, the presence of many functional groups, especially the phenolic hydroxyl group, is responsible for the antimicrobial effects of lignin. This functional group can modify the physiological process of the microorganism, leading to damaging the cell membrane and eventually causing the loss of functionalities of the microorganism [78,79]. It was suggested that the phenolics in lignin can disrupt the cell wall, resulting in altered physiological processes and, eventually, dysfunction of parts of the microorganism and growth inhibition [20].

#### 3.2.2. Antimicrobial Activities of SLNPs Incorporated into PBS Composite Films In Vitro

**Figure 7:**
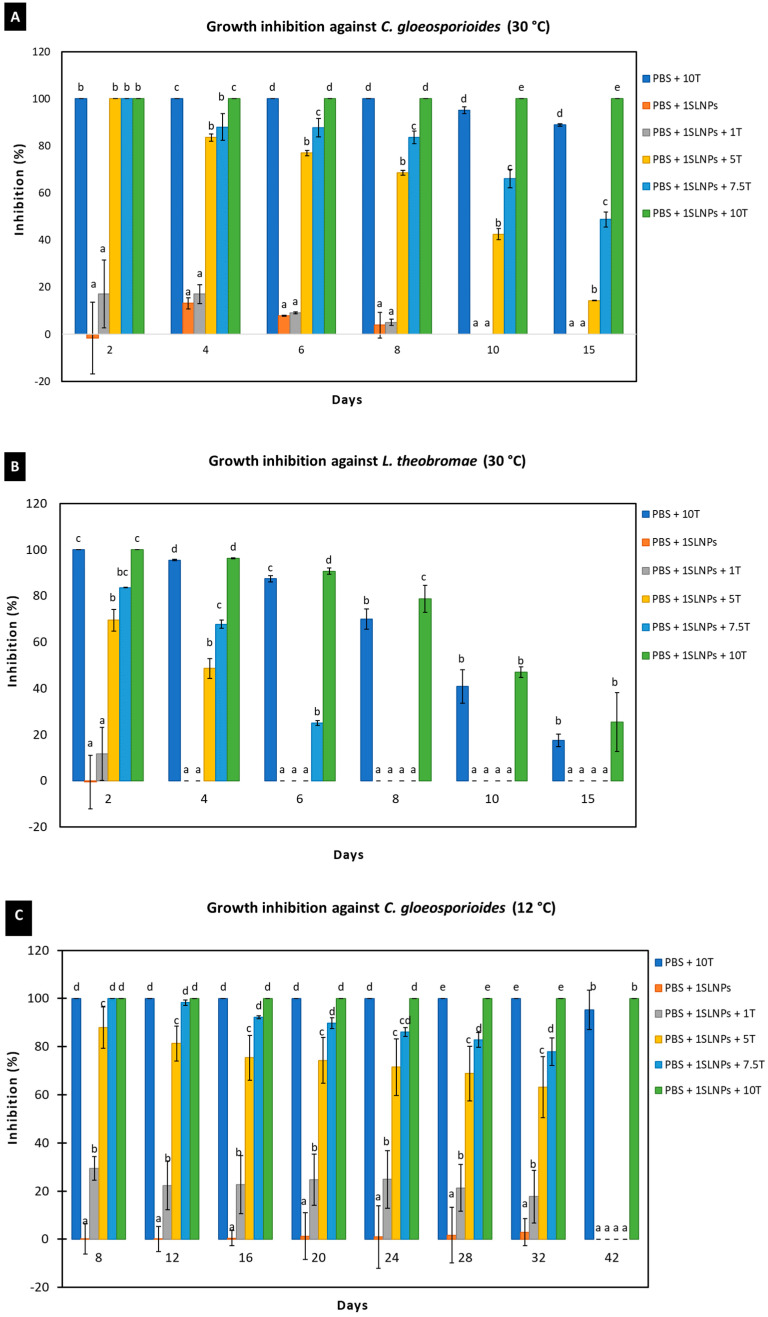

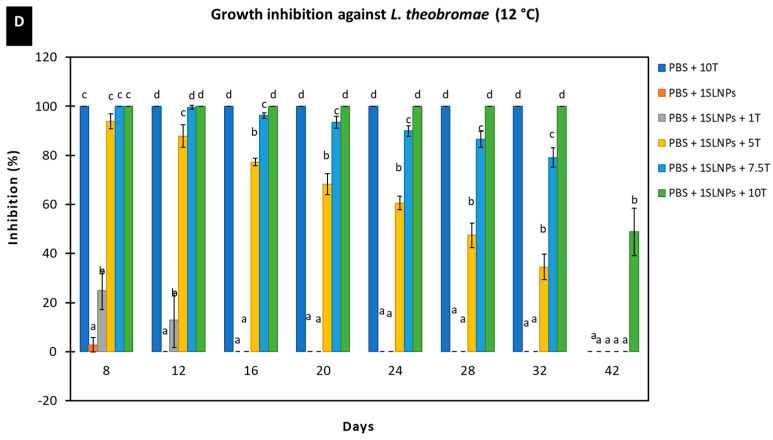
Growth inhibition of PBS composite films containing 1% SLNPs at 30 °C against (**A**) *C. gloeosporioides* and (**B**) *L. theobromae*; at 12 °C against (**C**) *C. gloeosporioides* and (**D**) *L. theobromae*. Values are presented as mean (*n* = 3) with the standard deviation represented by vertical bars. Means followed by the same letter between treatments per day are not significantly different at *p* ≤ 0.05 using DMRT.

Figure 7 shows the antimicrobial activities against *C. gloeosporioides* and *L. theobromae* of 1% SLNPs incorporated into the PBS composite films containing thymol, determined using the vapor diffusion method. Thymol can inhibit the mycelial growth of the tested fungi either at 30 or 12 °C, and the inhibition was significantly reduced as the concentration of thymol decreased. At 30 °C, 10% (*w*/*w*) thymol in PBS was sufficient to fully inhibit the growth of *C. gloeosporioides* for at least 8 days; the % inhibition decreased to 95.1 and 88.94% on days 10 and 15, respectively. However, with the presence of 1% SLNPs in the PBS + 10T, mycelial growth was not observed until the last day of observation, which equates to 100% growth inhibition. Meanwhile, both PBS + 10T and PBS + 1SLNPs + 10T fully inhibited the mycelial growth of *L. theobromae* until day 2. The % inhibition decreased over time with 95.44, 87.40, 69.91, 40.88, and 17.53% on days 4, 6, 8, 10, and 15, respectively, for PBS + 10T, and 96.21, 90.68, 78.77, 47.03, and 25.42%, respectively, for PBS + 1SLNPs + 10T. Although the calculated % inhibition in PBS + 1SLNPs + 10T was generally higher than in PBS + 10T, the significant (*p* ≤ 0.05) difference was determined starting on day 10 for *C. gloeosporioides* and on day 6 for *L. theobromae*. It is interesting to note that with the presence of 1% SLNPs, the antimicrobial effects against the studied fungal species increased, as well as the duration of effectiveness.

The observation for the growth inhibition against the tested fungi at 12 °C was started on day 8 because no significant (*p* > 0.05) mycelial growth was observed before that day. Like the observations at 30 °C, the inhibition against *C. gloeosporioides* and *L. theobromae* reduced as the concentrations of thymol decreased, but both PBS + 10T and PBS + 1SLNPs + 10T produced 100% inhibition against the tested fungi for 32 days. Since temperature directly affects fungal growth [80] and the fungal metabolic activities important for growth are slowed down at low temperatures, making the fungi dormant until optimum temperature [81], samples were moved out to ambient temperature (30 ± 2 °C) for further observation. After 10 days in ambient temperature, there was an average of 3.67 mm mycelial growth of *C. gloeosporioides* in PBS + 10T, and the calculated % inhibition was 95.24%; however, mycelial growth was still not observed in PBS + 1SLNPs + 10T. Thus, the inhibition remained at 100%. Meanwhile, for *L. theobromae*, the mycelia grew up to the edge of the Petri dishes after ten days in PBS + 10T, resulting in zero inhibition. However, an average of 39.42 mm mycelial growth was recorded in PBS + 1SLNPs + 10T, resulting in 48.81% growth inhibition. The results signify that the presence of 1% SLNPs in the PBS composite films containing thymol could significantly enhance the antimicrobial effects, particularly in the final days of observation. This could be due to the molecular interaction of SLNPs and thymol, particularly the substantial π-stacking between aromatic compounds in thymol and aromatic units of lignin [82], which could retain thymol in the PBS matrix for a longer period, leading to longer antimicrobial effectiveness.

#### 3.2.3. Antimicrobial Activities of PBS Composite Films In Vivo

**Figure 8:**
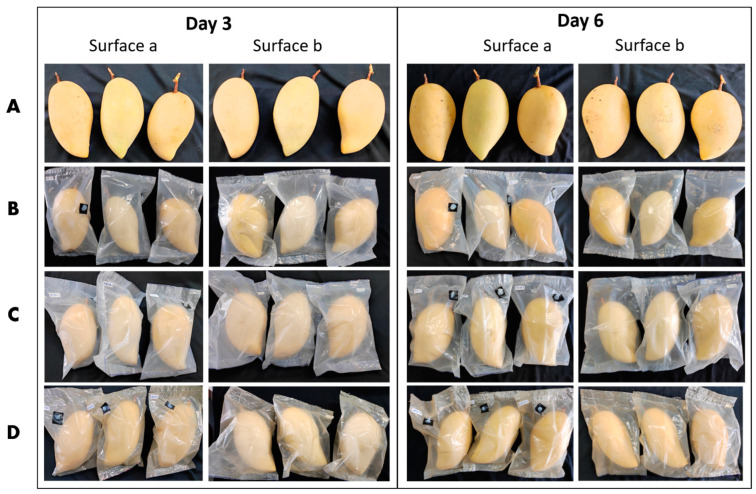
Mango fruit samples on days 3 and 6, stored at 12 °C and 90 ± 5% RH. (**A**) Control, (**B**) neat PBS, (**C**) PBS + 10T, and (**D**) PBS + 1SLNPs + 10T.

The antimicrobial activities on “Nam Dok Mai Si Thong” mango fruit in PBS composite films containing 1% SLNPs and 10% thymol were quantified by determining the total area of decay on the last day of observation, day 33. Mango samples were unpacked on this day due to the unmarketable appearance of control mangoes and samples packed in neat PBS. There were black-brown sunken circular spots observed on these mango samples, a manifestation of the mango disease anthracnose, caused by *C. gloeosporioides* [83]. Additionally, these fruit samples showed a dark-brownish area on the surface around the base of the fruit’s stem end, an indicator of stem-end rot disease caused by *L. theobromae* [84]. Conversely, no visible fruit diseases were observed in the early stage of storage, e.g., on days 3 and 6, as shown in Figure 8.

Table 5 is the summary of the calculated total decay of the mango samples. Mango fruit packed in neat PBS exhibited the largest total area of decay, with 17.2 cm^2^, followed by control samples, with 16.0 cm^2^. This could be due to the saturated RH inside the package (100% RH) compared to the % RH of the cold storage chamber (90 ± 5%). As shown in the work of Dannemiller et al. [85], the fungal growth rate at 100% equilibrium RH was higher than at 85% equilibrium RH. Meanwhile, 10% thymol in the PBS matrix significantly inhibited the growth of the decay-causing fungal species, as shown by the low total area of decay of 5.2 cm^2^. However, with the presence of 1% SLNPs in the PBS + 10T composite, the total area of decay was significantly (*p* ≤ 0.05) reduced further to 1.1 cm^2^. The results were associated with what was obtained in the in vitro study.

## 4. Conclusions

The incorporation of SLNPs into the PBS film containing thymol was shown to increase the tensile strength, barrier against oxygen, thermal decomposition temperature, and antifungal activities. FTIR results confirmed the good interactions among SLNPs, thymol, and PBS matrix, and SEM corroborated the homogeneity of the PBS composite film. The synergistic effects of SLNPs and thymol in the PBS matrix showed the strongest microbial growth inhibition against *C. gloeosporioides* and *L. theobromae*, two major fungal species that cause anthracnose and stem-end rot diseases in mango fruit, respectively. This work showed that SLNPs could be an attractive natural alternative to synthetic substances for enhancing polymer properties without compromising the biodegradability of the resultant material. Furthermore, the results gathered are important and recommended for extending the shelf life of many economically important fruit crops that are susceptible to anthracnose and stem-end rot diseases. In addition, this natural alternative could potentially be applied as an antimicrobial packaging for other food products.

## Figures and Tables

**Table 1:** PBS composite films’ formulations.

Formulations	PBS (*w*/*w*)	SLNPs (*w*/*w*)	Thymol (*w*/*w*)
PBS	100	-	-
PBS + 10T	90	-	10
PBS + 1SLNPs	99	1	-
PBS + 1SLNPs + 1T	98	1	1
PBS + 1SLNPs + 5T	94	1	5
PBS + 1SLNPs + 7.5T	91.5	1	7.5
PBS + 1SLNPs + 10T	89	1	10

PBS: polybutylene succinate; SLNPs: softwood kraft lignin nanoparticles; T: thymol.

**Table 2:** Thermal properties of neat PBS and PBS composites.

Film Samples	*T_g_* (°C)	*T_m1_* (°C)	*T_m2_* (°C)	*T_c_* (°C)	*T_d_^o^* (°C)	*T_d_^p^* (°C)	*X_c_* (%)
Neat PBS	−39.1	105.2	112.5	91.3	354.1	394.5	36.1
PBS + 1SLNPs	−35.7	105.2	112.7	90.8	364.7	405.0	38.5
PBS + 10T	−43.7	100.0	109.5	87.5	361.7	404.0	35.4
PBS + 1SLNPs + 1T	−33.2	104.8	112.8	90.3	364.6	405.5	34.1
PBS + 1SLNPs + 5T	−34.3	105.2	112.5	90.2	362.7	402.8	35.9
PBS + 1SLNPs + 7.5T	−40.9	104.5	112.5	90.0	362.9	400.2	37.0
PBS + 1SLNPs + 10T	−42.1	103.7	111.8	89.7	365.2	404.8	32.2

***T_g_***: glass transition temperature; ***T_m_***: melting temperature; ***T_c_***: crystallization temperature; ***T_d_****^o^*: onset of thermal decomposition temperature; ***T_d_^p^***: peak of thermal decomposition temperature; and ***X_c_***: crystallinity.

**Table 3:** Young’s modulus (YM), tensile strength (TS), and elongation at break (EB) in the machine direction of neat PBS and PBS composite films.

Film Samples	YM (MPa)	TS (MPa)	EB (%)
Neat PBS	593.4 ± 84.2 ^bc^	34.3 ± 1.4 ^bc^	11.6 ± 0.8 ^a^
PBS + 1SLNPs	601.9 ± 57.9 ^c^	35.6 ± 1.8 ^c^	10.3 ± 0.8 ^a^
PBS + 10T	491.9 ± 62.8 ^ab^	28.3 ± 2.5 ^a^	11.9 ± 0.4 ^a^
PBS + 1SLNP + 1T	589.1 ± 63.9 ^bc^	34.9 ± 1.2 ^bc^	11.6 ± 0.9 ^a^
PBS + 1SLNP + 5T	445.3 ± 97.3 ^a^	34.0 ± 7.1 ^bc^	11.6 ± 2.1 ^a^
PBS + 1SLNP + 7.5T	466.5 ± 62.2 ^a^	34.9 ± 3.2 ^bc^	11.2 ± 1.7 ^a^
PBS + 1SLNP + 10T	486.6 ± 104.4 ^ab^	30.0 ± 4.6 ^ab^	10.6 ± 0.6 ^a^

Values are presented as mean ± standard deviation (*n* = 5). Means followed by the same superscript letters in columns are not significantly different at *p* ≤ 0.05 using DMRT.

**Table 4:** Thickness, OP, and WVP of neat PBS and PBS composites films.

Film Samples	Thickness (µm)	Oxygen Permeability (cm^3^ m h^−1^ m^−2^ atm^−1^)	Water Vapor Permeability (g m h^−1^ m^−2^ atm^−1^)
Neat PBS	29.8 ± 0.8 ^a^	2.28 × 10^−3^ ± 0.0002 ^b^	1.54 × 10^−3^ ± 0.0001 ^d^
PBS + 1SLNPs	30.0 ± 1.6 ^a^	1.59 × 10^−3^ ± 0.0002 ^a^	1.41 × 10^−3^ ± 0.0001 ^cd^
PBS + 10T	31.8 ± 4.9 ^a^	2.37 × 10^−3^ ± 0.0002 ^bc^	1.14 × 10^−3^ ± 0.0001 ^ab^
PBS + 1SLNP + 1T	30.2 ± 2.7 ^a^	2.40 × 10^−3^ ± 0.0004 ^bc^	1.28 × 10^−3^ ± 0.0001 ^bc^
PBS + 1SLNP + 5T	30.4 ± 2.7 ^a^	2.08 × 10^−3^ ± 0.0001 ^b^	1.15 × 10^−3^ ± 0.0002 ^ab^
PBS + 1SLNP + 7.5T	33.4 ± 1.5 ^a^	2.56 × 10^−3^ ± 0.0005 ^bc^	1.28 × 10^−3^ ± 0.0003 ^ab^
PBS + 1SLNP + 10T	33.4 ± 2.4 ^a^	2.84 × 10^−3^ ± 0.0004 ^c^	1.09 × 10^−3^ ± 0.0000 ^ab^

Values are presented as mean ± standard deviation (*n* = 4). Means followed by the same superscript letters in columns are not significantly different at *p* ≤ 0.05 using DMRT.

**Table 5:** Total area of decay on day 33.

Treatment	Decay Area (cm^2^)
CONTROL	16.0 ± 2.1 ^c^
Neat PBS	17.2 ± 0.3 ^c^
PBS + 10T	5.2 ± 0.7 ^b^
PBS + 1SLNPs + 10T	1.1 ± 0.1 ^a^

Means followed by the same superscript letter between treatments are not significantly different at *p* ≤ 0.05 using DMRT.

## Data Availability

All data are contained in the article.
